# From Benchtop to Desktop: Important Considerations when Designing Amplicon Sequencing Workflows

**DOI:** 10.1371/journal.pone.0124671

**Published:** 2015-04-22

**Authors:** Dáithí C. Murray, Megan L. Coghlan, Michael Bunce

**Affiliations:** Trace and Environmental DNA Laboratory, Department of Environment and Agriculture, Curtin University, Perth, Western Australia, Australia; Natural History Museum of Denmark, DENMARK

## Abstract

Amplicon sequencing has been the method of choice in many high-throughput DNA sequencing (HTS) applications. To date there has been a heavy focus on the means by which to analyse the burgeoning amount of data afforded by HTS. In contrast, there has been a distinct lack of attention paid to considerations surrounding the importance of sample preparation and the fidelity of library generation. No amount of high-end bioinformatics can compensate for poorly prepared samples and it is therefore imperative that careful attention is given to sample preparation and library generation within workflows, especially those involving multiple PCR steps. This paper redresses this imbalance by focusing on aspects pertaining to the benchtop within typical amplicon workflows: sample screening, the target region, and library generation. Empirical data is provided to illustrate the scope of the problem. Lastly, the impact of various data analysis parameters is also investigated in the context of how the data was initially generated. It is hoped this paper may serve to highlight the importance of pre-analysis workflows in achieving meaningful, future-proof data that can be analysed appropriately. As amplicon sequencing gains traction in a variety of diagnostic applications from forensics to environmental DNA (eDNA) it is paramount workflows and analytics are both fit for purpose.

## Introduction

The myriad of names and acronyms associated with high-throughput DNA sequencing (HTS) is undeniably impressive and the number of applications for which the technology itself has proven useful equally matches this. To date, amplicon sequencing [1], whereby PCR products are generated, converted to libraries, pooled and then sequenced, has been the method of choice in many HTS studies. Amplicon sequencing has been used in, or proposed for, a wide range of contexts that include, amongst others, biomonitoring [2–7], diet analysis [8–13] and bacterial metagenomics [14–20]. As a result of the ease with which the technology can be applied across an array of disciplines, it can at times prove to be a minefield for scientists seeking to avail of it. This is especially true for those with limited experience in either wet-lab molecular biology skills or computational bioinformatics. The latter of these areas has received much attention; the importance of the former is often under-appreciated.

Currently, most primary literature, reviews and opinion articles surrounding HTS tend to focus on the applications of the technology [3,5,6,9,21–24], platform evaluations [25,26] and bioinformatic approaches to data analysis [27–33]. While all three are extremely important in the generation of high fidelity data, a heavy focus on these aspects fails to address the need to pay close attention to the implementation of protocols and procedures at the bench. The data one has to work with is, and will only ever be, as good as the quality of experimental procedures implemented and no amount of high-end bioinformatics can compensate for poorly prepared samples, artefacts or contamination. It is therefore imperative that careful consideration is given to the ways in which samples are screened for sequencing, in addition to the method used to generate the amplicon sequencing library. These aspects are independent of the equally important need to carefully choose extraction methods that are optimised for the chosen substrates. While DNA isolation methods are a key consideration, this is dealt with extensively elsewhere. Instead, this paper focuses on how best to approach amplicon workflows following DNA extraction to generate robust and representative datasets for a given DNA isolation.

Through a series of simple experiments (Table 1), various aspects that should be considered when preparing to embark on the use of amplicon sequencing are highlighted, some aspects of which are equally as applicable to shotgun sequencing. These experiments focus primarily on three areas of experimental design or benchwork within the typical amplicon sequencing workflow: sample screening, the target region, and library generation. Finally, although not a focus of the paper, certain pertinent considerations in relation to data analysis that are seldom acknowledged in other literature will also be addressed. It is hoped that the following may address the distinct lack of literature in relation to sample preparation and library generation. It is advocated that closer attention is required at the bench when conducting amplicon sequencing. Ultimately, it may be appropriate to define a set of flexible guidelines, such as the MIQE guidelines used for qPCR data [34], for the reporting of amplicon data generation and analysis.

**Table 1:** Details for the experiments conducted.

Experiment	Purpose	Methods	Results
**Experiment 1: Importance of sample screening**	Illustrate the importance of quantifying samples using a dilution series to select an appropriate working dilution free of inhibition containing a sufficient quantity of input template DNA	**Main: 2.2.1** *(see also*: *Section 2*.*1*.*1*. S1A Fig *&* S1 Table)	Section 3.1. Fig 2
**Experiment 2: Assessing the amplicon target region**	Explore the potential benefits to the downstream processing of high-throughput sequencing data arising from the inclusion of amplicon-specific single-source samples embedded into sequencing runs	**Main: 2.2.2** *(see also*: S1B Fig *&* S1 Table)	Section 3.2. Fig 3
**Experiment 3: Importance of experimental controls**	Demonstrate the importance of control reactions in bacterial metagenomics and other fields using samples with a high propensity for environmental contamination	**Main: 2.2.3** *(see also*: S1C Fig *&* S1 Table)	Section 3.3. S2 Table
**Experiment 4: Library generation efficiency**	Assess the efficiency drop-off associated with the use of fusion tagged primers of different ‘architecture’ when compared to standard non-fusion tagged template specific primers	**Main: 2.2.4** *(see also*: *Section 2*.*1*.*1*. S1D and S1E Fig *&* S1 Table)	Section 3.4. S3 Table
**Experiment 5: Analysis parameters and their impact**	Highlight the difficulties in choosing appropriate quality and abundance filtering parameters when analysing complex, heterogeneous samples; the composition of which are unknown.	**Main: 2.2.5** *(see also*: Fig 1, S1F Fig *&* S1 Table)	Section 3.5. Fig 4, S4 Table

The purpose of each numbered experiment is shown in addition to the title used for each one in the methods and results section. The appropriate methods sections, results sections and figures to consult for each experiment are also given.

## Materials and Methods

Some of the following methodologies were specifically designed for this study; others have utilised samples and/or data drawn from previous studies [24,35–38]. The materials and methods below provide an overview of the methodologies and the reader is referred to the original publications and also the supplementary online information where schematics of all experiments conducted are presented (S1A–S1F Fig). Each of four important steps in amplicon workflows: sample screening (Section 2.2.1 and S1A), the target region (Section 2.2.2 and S1B Fig), library generation (Sections 2.2.3, 2.2.4 and S1C–S1E Fig) and data analysis (Section 2.2.5 and S1F Fig), is addressed separately in the materials and methods that follow. General methods employed during sample screening, amplicon generation, DNA sequencing and data analysis that were common to all areas are detailed first (Section 2.1) before more focused information on each of the four aforementioned steps (Section 2.2). Any further detailed information on the samples or experimental workflows used is available in previous publications [24,35–38] or from the authors upon request. Where applicable amplicon sequence reads have been uploaded to Data Dryad (doi:10.5061/dryad.2qf0t).

### 2.1. General methods

#### 2.1.1. DNA extraction and screening

A variety of samples and extraction methods are used throughout these experiments. Extraction protocols followed can be found in the original publications where indicated [24,35–38], but typically involved silica-based purification methods to isolate DNA. Where sample extraction has not been reported previously, the details of the extraction procedure are found below in Section 2.2.

All samples used were screened to determine the appropriate working dilution containing sufficient DNA free of inhibition using quantitative PCR (qPCR) on a SYBR-based STEP-ONE Applied Biosystems Real-Time PCR instrument [35,39]. Samples were assessed based on Cycle Threshold (C_T_) values, curve form and melt-curves. Extraction controls were conducted for each batch of extractions and screened using qPCR to test for contamination arising from laboratory practice, reagents, or the environment. If positive for the presence of DNA, extraction controls were included in tagged qPCR assays (see Section 2.1.2). All qPCR reaction conditions and reagent components can be found in previous publications where indicated below, and primer details can be found in S1 Table. Details are provided below for any qPCR reactions not previously reported.

#### 2.1.2. Amplicon generation and sequencing

For samples deemed to have sufficient DNA copy number and determined to be free of inhibition, amplicon sequences were always generated in triplicate via qPCR using a unique combination of forward and reverse Multiplex Identifier (MID-) tagged (i.e. indexed) primers [27,40] (for the only exceptions to this see Section 2.2.1 and S1A Fig). For each tagged qPCR assay, negative reaction controls were included and, if found to contain amplifiable DNA, were incorporated into the appropriate sequencing library. Resultant amplicon products were purified following the Agencourt AMPure XP PCR Purification Kit protocol (Beckman Coulter Genomics, NSW, Aus.) and were eluted in 40 μL of Ultrapure H_2_O. Purified amplicon products for each sequencing library for each platform were electrophoresed on ethidium bromide stained 2% agarose gel and pooled in equimolar ratios based on band intensity to form sequencing libraries.

In order to determine an appropriate volume of library for sequencing, each amplicon library was serially diluted and quantified using qPCR against a serial dilution of a custom synthetic oligonucleotide of known molarity. Reaction components and conditions were the same for each sequencing platform with the exception of platform specific primers appropriate to the sequencing adaptors. Each 25 μL reaction contained 2X ABI Power SYBR master mix (Applied Biosystems, CA, USA), 0.4 μM each of platform specific forward and reverse primer (IDT), and 2 μL of pooled library. Each reaction underwent the following cycling conditions: 95°C for 5 mins; 40 cycles of 95°C for 15 s, 56°C for 1 min followed by a 1°C melt curve. All sequencing was conducted according to manufacturer’s protocols using one of three sequencing platforms: GS Junior (Roche), Ion Torrent PGM (Life Technologies) and MiSeq (Illumina). Sequencing on Roche was conducted using LibA chemistry. Ion Torrent PGM emulsion PCR (emPCR) was conducted on a OneTouch2 using 400bp chemistry and sequencing was performed on 314 chips. Finally, Illumina MiSeq sequencing used V2 300 cycle chemistry on nano flow cells. To enable direct comparisons both PGM and MiSeq used single direction sequencing only, despite the fact that paired-end sequencing is available in the latter.

#### 2.1.3. Data analysis

**Fig 1:**
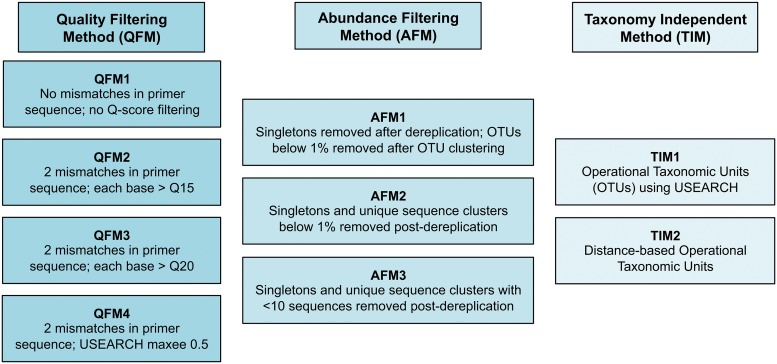
Definitions used in assessing the importance of analysis parameters. Shown are the definitions for quality and abundance filtering methods used in assessing their impact on both the number of operational taxonomic units (OTUs) and distance-based operational taxonomic units (DTUs) [24] obtained for a given sample. maxee—Maximum Expected Error

Regardless of the platform, amplicon sequence reads were deconvoluted in Geneious v7.1.3 (this version of Geneious is used throughout this paper) [41] based on unique primer indexes. As a first step in deconvolution any sequences found to contain ambiguous base calls (e.g. N) were discarded. Identification tags and primer sequences were trimmed from all reads in Geneious, allowing for no mismatch in either length or base composition as a means of quality filtering, using the inbuilt “Separate Reads by Barcode” and “Trim Ends” functions respectively. The only exception to this can be found in Section 2.2.5 where in some instances two base mismatches in the primer sequences were allowed (see also Fig 1 and Section 2.2.5). Unless otherwise stated in Section 2.2, Quality Score (Q-Score) filtering was not performed. Sequences were subsequently dereplicated at 100% identity across their full length using USEARCH v7 (this version of USEARCH is used throughout this paper) [42,43], and low abundant sequence clusters, defined as those below 1% of the total number of unique sequences, were removed using USEARCH also. Dereplicated sequences were clustered at a 97% threshold using the UPARSE [43] algorithm implemented in USEARCH. Chimeric sequences were also identified and removed using USEARCH [42,44]. At all stages of dereplication and OTU clustering abundance information was retained and used when calculating taxa/sequence abundance or error rates. Where appropriate, sequences were queried against the NCBI GenBank nucleotide database [45] using BLASTn [46] in YABI [47], enabling taxonomic identification. Sequences were searched without a low complexity filter, with a gap penalties existence of five and extension of two, expected alignment value less than 1e-10 and a word count of seven. The BLASTn results obtained were imported into MEtaGenome ANalyzer v4 (MEGAN) [32], where they were mapped and visualised against the NCBI taxonomic framework (min. bit score = 35.0, top percentage = 5%, min. support = 1). In cases where taxonomic identification was necessary, a genus or family level assignment of a query sequence was required to have a BLASTn percentage similarity to a reference sequence of 97% or 95% respectively. Instances where data analysis deviated from the above steps are detailed where necessary below.

### 2.2. Specific methodologies

#### 2.2.1. Experiment 1: Importance of sample screening

To evaluate the importance of screening samples for inhibition and low target template amount, an environmental faecal sample was obtained from a *Eudyptula minor* (Little Penguin) individual. DNA was extracted from the faecal sample, serially diluted, and screened via qPCR as described in Murray et al. [35] using 16S1F/16S2R degenerate fish primers [48] (see also S1A Fig and S1 Table). An appropriate working dilution of the sample deemed to have sufficient DNA copy number and free of inhibition (see Section 2.1.1) was used for sequencing on both the Ion Torrent PGM and GS Junior. In addition to this, both an aliquot of the working dilution spiked with an extremely inhibited soil DNA extract, to mimic inhibition, and a dilution classed as “Low Template” were selected for sequencing. For each sample, the detection and percentage abundance of two baitfish genera, *Sardinops* (specifically *S*. *sagax*—Australian pilchard) and *Engraulis* (specifically *E*. *australis—*Australian anchovy) were examined. The former being in the highest abundance: the latter in lowest abundance, as determined by a taxon-specific qPCR assay (see S1 Table and [35]).

The handling of the penguin, and the collection and use of the faecal sample was conducted by experienced handlers under a strict set of animal ethics guidelines approved by the Murdoch University Animal Ethics Committee (permit no. W2002/06) as part of a long-term study into *Eudyptula minor* (Little Penguin) diet. Faecal sampling and DNA extraction were performed as part of a previously published study [35] and not as a part of this study, however ethics approval covers the use of the faecal sample DNA extract in this study.

#### 2.2.2. Experiment 2: Assessing the amplicon target region

Five single-source bird tissue samples were used to assess error profiles associated with a specific amplicon target region (see S1B Fig). *Calyptorhynchus latirostris* (Carnaby’s Black Cockatoo) and *C*. *lathami* (Glossy Black Cockatoo) samples were collected, and DNA extracted, as detailed in White *et al*., 2014 [38]. Tissue samples of *Gallus gallus* (Chicken), *Dromaius novaehollandiae* (Emu) and *Struthio camelus* (Ostrich) were bought commercially and DNA was extracted using a Qiagen DNeasy Blood and Tissue Kit following the manufacturer’s protocol. For each sample an approximately 250 bp region of the mitochondrial 12S rRNA gene was amplified and MID-tagged using 12SA/H avian primers (see S1 Table and [49,50]) via qPCR (reaction components and conditions as detailed in [24]), and then sequenced on both Ion Torrent PGM and Illumina MiSeq platforms.

Amplicon sequence reads for each bird were randomly sub-sampled a total of 25 times to a depth of 1,000 sequences using seqtk (available from https://github.com/lh3/seqtk) following deconvolution into sample batches (see Section 2.1.3). Each sub-sample was dereplicated at 100% identity to determine the most abundant sequence, with the abundance of each unique sequence appended to sequence names for use in calculating error rates. The most abundant sequence was taken as the reference sequence. For both platforms the most abundant sequence was identical thus meaning it is likely ‘correct.’ Each set of sub-sampled sequences was individually aligned using MUSCLE with default parameters [51]. Alignments were imported into excel and for each sample the error associated with each base was calculated as a percentage of the total number of non-dereplicated sequences that differed from the reference sequence at that specific base. This was performed using an in-house macro; the output of which can be seen in S1 File. The error associated with each sub-sample was subsequently calculated as the mean error across all bases. The overall percentage error rate for each bird species on both the Ion Torrent and MiSeq was taken as the mean error rate across all 25 sub-samples of each species.

The collection and use of DNA material from Cockatoos was approved by, and conducted under, Department of Parks and Wildlife (Western Australia) scientific purposes licences SC000357, SC000920, SC001230, Australian Bird and Bat Banding Authority 1862 and Animal Ethics Committee approvals DEC AEC 11/2005 and 32/2008 held by P. R. Mawson. Samples of Chicken, Emu and Ostrich (all non-endangered) were purchased from Franks Gourmet Meats, Perth, WA, Australia, and are exempt from a collection permit.

#### 2.2.3. Experiment 3: Importance of experimental controls

To illustrate the importance of control reactions in bacterial metagenomics and other fields dealing with samples with a high likelihood of environmental contamination, bacterial 16S data from hair samples were generated and analysed as detailed in Tridico *et al*., 2014 ([37], see also S1C Fig and S1 Table). Briefly, pubic and scalp hair were self-sampled by male and female volunteers. Hair samples were prepared and extracted as detailed in Tridico *et al*., 2014. Samples were screened using Bact_16S_F515 and Bact_16S_R806 primers [52,53] and amplicon libraries were generated, sequenced and analysed as per Tridico *et al*., 2014.

The collection of human hairs for bacterial profiling was approved by, and conducted in accordance with, Murdoch University Human Research Ethics Committee Policies and Guidelines (Project Number 2011/139). Each volunteer was made aware of the nature of the study and gave written, informed consent. Hairs were self-collected from two somatic origins and placed in sample bags bearing no information that would allow the identification of any individual participant in the study [37].

#### 2.2.4. Experiment 4: Library generation efficiency

Quantitative PCR using the plant plastid trnLg/h primer set [54] was carried out to investigate the issues surrounding efficiency drop-off associated with the use of “full” fusion tagged primers (see S1D Fig and S1 Table), i.e. those with MID tags and sequencing adapters upstream of the template specific primer (TSP) (see S1E Fig and [40]). A single-source plant extract in addition to two complex, heterogeneous Traditional Chinese Medicines (TCM) were used; a MoBio Plant DNA Isolation kit was used following the manufacturer’s protocol for the single-source plant sample DNA extraction, while sampling and extraction of TCMs are detailed in Coghlan et al. [36]. Each sample was amplified in triplicate using either (1) standard non-fusion TSP; (2) MID encoded TSP (3) “full” fusion tagged TSP or (4) “full” fusion tagged TSP with standard non-fusion TSP spiked in (see S1D and S1E Fig). For (1–3) each qPCR reaction was carried out in a total volume of 25 μL containing 2X ABI Power SYBR master mix (Applied Biosystems, CA, USA), 0.4 μM each of the appropriate forward and reverse TSP (IDT) and 2 μL DNA extract. For (4) the previous components were also used but an additional 0.04 μM spike-in of each the forward and reverse standard non-fusion TSP (IDT) was also used. For each reaction C_T_ threshold was set at 0.1.

TCM samples were obtained from, and approved for use by, the Wildlife trade section of the Department of Sustainability, Environment, Water, Population and Communities (Australia) after being seized by Australian Customs and Border Protection Service at airports and seaports across Australia. The samples were seized because they contravened Australia's international wildlife trade laws as outlined under Part 13A of the Environment Protection and Biodiversity Conservation Act 1999 (EPBC Act). The samples were stored in a quarantine-approved facility within the laboratory after being catalogued. The samples were patent medicines available over the counter and were donated by Australian Customs and Border Protection Service under no ethics or quarantine requirements and were deemed suitable to be used for specific and general research purposes by the Customs service [36].

#### 2.2.5. Experiment 5: Analysis parameters and their impact

To demonstrate the variability in calculated OTU (operational taxonomic unit) diversity within a sample, a single bulk-bone sample, comprising ~50 individual bones and containing an unknown number of taxa, was extracted and screened using the 16Smam1 and 16SMam2 mammalian specific primer set [55]. Amplicon sequences were generated for short sections within the mammalian mitochondrial 16S gene using the 16Smam1 and 16SMam2 primer set and sequenced using the Ion Torrent PGM as described in Murray *et al*., 2013 [24] (see also S1F Fig and S1 Table). After deconvolution following the method detailed in 2.1.3 the data were analysed using various quality filtering methods (QFM), abundance filtering methods (AFM), and taxonomy-independent methods (TIM) of diversity analysis as shown in Fig 1. Quality Score filtering was conducted in Galaxy [56–58] using the FASTQ Quality Filter tool. Maximum expected error (maxee) quality filtering, set at 0.5, was conducted using the fastq_filter command in USEARCH. Summary quality statistics were calculated in excel using fastq files post quality filtering for QFM1 and QFM4, prior to any further abundance filtering. Dereplication and OTU clustering at 97% was conducted using USEARCH also. DTU’s were determined post OTU clustering as described in Murray *et al*., 2013. Briefly, for DTU analyses, OTU’s were aligned using MAFFT [59] and alignments imported into MEGA v6.06 [60] where a distance matrix was created and exported. To determine OTU’s that differed from each other by less than 3% distance matrices were analysed in excel using an in-house macro, an example output of which is shown in S2 File. [24]. The impacts of DNA preservation, DNA degradation, mode of bone accumulation and deposit setting will have negligible impact on the results of this experiment as the exact same set of amplicon sequences, from the exact same DNA extract, are used for each combination of QFM, AFM and TIM used. The dataset in this experiment is therefore static throughout and any biases introduced by any of the aforementioned factors will be the consistent across all methods.

## Results and Discussion

Much attention has been devoted to the bioinformatic challenges associated with the analysis of amplicon sequencing data. There are a suite of programs, tools and pipelines available to assist in the deconvolution, filtering and parsing of data. As a relatively new field there is no obvious consensus on how data should, or should not, be handled bioinformatically, with the exception that sequence clusters in very low abundance should be filtered. Likewise there is no consensus on what is best-practice for data generation. Arguably the importance of data generation has taken a backseat to the computational workflows that surround bioinformatics. Bioinformaticians, rightly so, ask key questions of researchers with regard to replicates, coverage and filtering. They are less likely to ask questions about input copy number, PCR inhibition, contamination and the appropriateness of benchtop protocols. This study, through the presentation of new and existing empirical data, seeks to demonstrate the importance of both benchwork and bioinformatics. The purpose of this study is to raise awareness of potential pitfalls associated with amplicon-based workflows. The workflows dealt with in this paper do not include the process of actual DNA extraction, itself undeniably important, as this has been dealt with extensively elsewhere. The workflows presented here take as their starting point a working, amplifiable DNA extract, which can only be achieved through the careful consideration of both the scope of the project and type of substrate.

### 3.1. Experiment 1: Importance of sample screening

**Fig 2:**
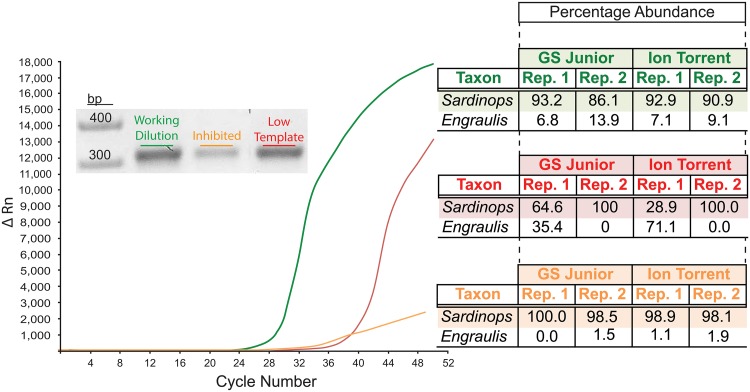
Quantitative PCR and sequencing results of the sample screening assay. Quantitative PCR curves indicating the presence of DNA and the degree of inhibition **(LEFT)** with agarose gel electrophoresis clearly indicating the presence of DNA post amplification via means of strong bands **(INSET ON GRAPH)**. Samples were subsequently sequenced and the percentage abundance of two fish genera is indicated, where, based on taxa-specific quantitative PCR results, *Sardinops* (specifically *S*. *sagax*—Australian pilchard) should be in the highest abundance, with *Engraulis* (specifically *E*. *australis—*Australian anchovy) being in the lowest abundance. **(RIGHT)**.

Adequate screening of samples prior to sequencing is an important task, yet fails to be routinely implemented in amplicon workflows. It is particularly prudent to assess the quality of samples when dealing with complex, heterogeneous substrates that may contain a variety of taxa or when examining samples that may contain highly degraded or low copy number DNA. There are arguably two primary factors that should be considered when evaluating samples for sequencing: the extent of inhibition, and the number of target input DNA template molecules used in generating an amplicon sequencing library. Both inhibition and low template number can have a negative impact upon the results obtained from amplicon sequencing workflows and failure to account for both can exacerbate other biases associated with amplicon sequencing. Common methods of screening samples include quantitative PCR (qPCR) and PCR end-point assays such as gel electrophoresis or capillary electrophoresis (e.g. Agilent Bioanalyzer). The advantage of using qPCR over end-point electrophoresis lies in the fact that it is easy to determine whether or not a sample is inhibited through the analysis of the Cycle Threshold (C_T_) values in a dilution series and the resultant curves. Traditional end-point assays such as electrophoresis are a blunt binary-state tool to assess inhibition and low-template samples; both will still produce bands on a gel (see gel image in Fig 2) or peaks on a Bioanalyzer trace. A case is not being made that samples should not be subjected to electrophoretic analysis, as this is a useful means for determining the presence of PCR artefacts. Rather, it would be practical to consider the additional use of qPCR or other similar methods of quantification (e.g. digital PCR), to assess the levels of inhibition and the absolute, or relative, number of target template molecules that are the input for amplicon sequencing workflows.

In a simple experiment involving the detection of two genera of fish, *Sardinops* (specifically *S*. *sagax*—Australian pilchard), in high abundance, and *Engraulis* (specifically *E*. *australis—*Australian anchovy), in low abundance, the effects of not being cognisant of inhibition or low DNA copy number are clearly demonstrated. When an appropriate working dilution exhibiting a sufficient number of input template copies and deemed free of inhibition (as determined by qPCR), was sequenced both fish species were detected in all PCR replicates, across two platforms (Fig 2, green line and shaded table). Furthermore, *Sardinops* was consistently detected as the fish species in the greater sequence abundance. In the case of the inhibited aliquot (Fig 2, orange line and shaded table) *Sardinops* was detected in all replicates and across both platforms, however *Engraulis* was not, and in those instances where it was detected it was typically at abundances <1%. When the low-template sample dilutions (Fig 2, red line and shaded table) were sequenced a similar pattern was observed, with again *Sardinops* being detected in all replicates and across both platforms and *Engraulis* being detected in only a few (see [4] for a further example of the non-detection across multiple replicates of a target species known to be in a sample). In this instance, the abundances were vastly different between the replicates and in one instance *Engraulis* appeared to be the fish species in the highest abundance.

The inclusion of PCR and/or sequencing replicates is without doubt an important aspect of any amplicon workflow serving to improve confidence and reliability in data interpretation ([61,62] although see [63]). Efforts have been made to determine the optimum level of PCR replicates, but it is acknowledged that the degree of replication required is dependent on the complexity of the sample in question and the objective of the study [61]. Additionally, it is also clear that simply increasing the depth of sequencing does not necessarily translate into an increased ability to detect low abundant taxa. In this study the increase in sequence depth afforded by the Ion Torrent did not improve *Engraulis* detection success. Arguably an extremely important, yet somewhat overlooked, aspect in generating an accurate species profile contained within any given sample is paying close attention to template input amount and quality, i.e. the level of amplifiable DNA and the degree of inhibition. This is becoming increasingly important as research efforts are moving towards quantitative interpretations of sequence abundance. Simply replicating PCRs using poor quality extracts is a blunt means of increasing the fidelity of amplicon sequence data.

It is acknowledged that PCR bias can greatly skew amplicon sequencing workflows [64–66], this is especially true when little or no attention is paid to input template amount or a sample’s amplifiable limits. Although only a small-scale experiment, the above serves to illustrate the importance of screening samples prior to sequencing (Fig 2). Amplicon sequencing results can clearly be obtained with low-template and inhibited samples but the reproducibility of these results is questionable: even more so if they are subsequently used in weighted analyses. Even when not interested in the relative abundance of taxa, OTUs or sequence variants, it is still nonetheless useful to screen samples for inhibition and low template amounts, as both of which can increase the possibility of false negatives. Whilst the absence of something in a sample can never truly be proven, being aware of the level of inhibition inherent within a sample or an estimate (however crude) of relative input can greatly improve the confidence surrounding presence, possible absence and/or abundance conclusions based off amplicon data. A common theme in the literature, including work by the authors, is to report the number of amplicon sequence reads obtained, but in reality a much more useful metric is to state the relative or absolute number of target templates provided to the reaction per replicate. In other words sequencing coverage is often a meaningless statistic—a PCR reaction that starts off a single molecule being the case in point. An increase in the use and reporting of quantitative data in amplicon workflows using qPCR or digital PCR can only assist in data fidelity and meaningful downstream analyses.

### 3.2. Experiment 2: Assessing the amplicon target region

**Fig 3:**
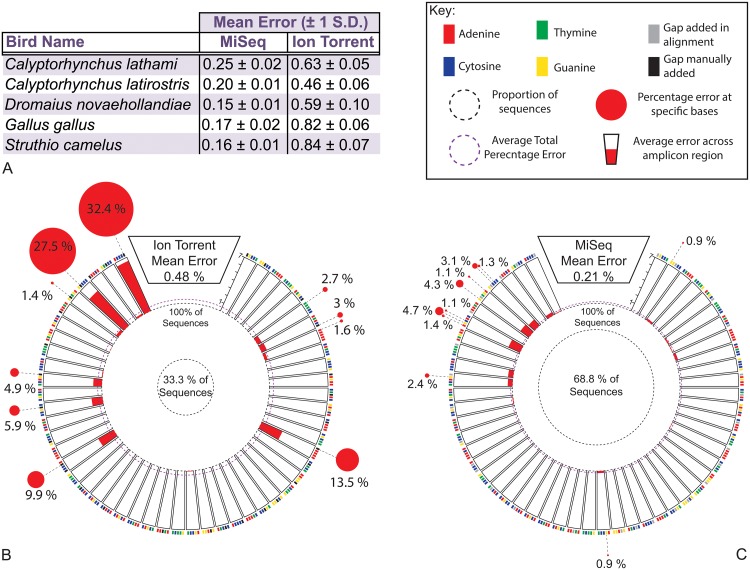
Average sequencing error rates across a single amplicon region. Average sequencing error rates are shown for multiple bird species across the whole of a short 12S rRNA gene region **(A)**. Additionally, the error profile across the gene region is shown for *Calyptorhynchus lathami* for both the Ion Torrent PGM **(B)** and MiSeq **(C)** with key. The error patterns observed were similar across all species sequenced. Error rates are shown across 5 bp segments and where error rates were above 1% for a single base this is indicated through the red circles.

Irrespective of the gene region chosen for investigation it is advisable to be aware of the composition of that region. This holds true especially for methods that rely on a small amount of data from the target region to infer conclusions, such as SNP data or taxonomic assignments between closely related taxa based off a few nucleotides. The primary reason for such attention is due to the fact that not all gene regions are “created” equal. Some gene regions can be more prone to error due to the occurrence of homopolymer stretches or secondary structures within the target area, particularly when dealing with 454 or Ion Torrent data. There are also well-recognised issues with quality and fidelity when dealing with target regions that are GC rich [67–70]. Both of these issues are in addition to the typical drop off in sequence quality and increase in potential error observed towards the sequencing length limitations of any given platform. The error rate, in addition to the quality of an amplicon sequence, is not uniform across the length of itself (Fig 3) nor is there necessarily a common error rate across different amplicon targets. Also worth noting is the potential for error rates to fluctuate between runs on the same platform on the same control DNA.

Some amplicon regions will undoubtedly sequence better than others due to the presence or absence of homopolymer regions and the complexity of the base composition. Rather than relying on generic error rates reported by the manufacturers or in the literature in the case of amplicons it is preferable to determine the error rate for the target region. In a small-scale experiment where single source samples for multiple bird species were sequenced, the error profile of the chosen ~250 bp target region of the 12S gene can be seen (Fig 3). It is evident that on both platforms the overall error varies slightly from species to species, yet a much greater range of mean error rates is observed in the case of the Ion Torrent PGM relative to MiSeq sequencing (Fig 3A). The variation in error rates observed across species is likely due to overall error rates associated with each platform. In addition to this it is observed that the percentage error for certain regions and specific bases far exceed the reported error rates cited for the platforms and in some cases, most notably with the Ion Torrent, certain regions recorded error rates as high as 7% (Fig 3B and 3C). Moreover, the increased error beyond that reported for the platform, and in some instances greater than 1%, often cited as a level used to eliminate erroneous sequences, is not solely confined to the 3’ end of the amplicon read. In the case of the Ion Torrent an error rate of 13.5% was calculated just 80 bases into the amplicon read (Fig 3B). Although significantly lower error rates at specific bases and in specific regions was observed in the MiSeq, bases and regions recording error rates approaching the 1% mark were found mid-way through the amplicon. In both cases this is despite average error rates for those sub-sampled sequences being calculated as 0.48% for Ion Torrent and 0.21% for the MiSeq (Fig 3B and 3C). The propensity for error is again highlighted in the case of the Ion Torrent whereby only 33.3% of sequences obtained for that sub-sample were contained within the highest unique cluster, which is alarming given that it is a single source sample, with theoretically only one possible sequence composition, yet two thirds of the sequences differed from the most common. Although the error profile for only one sub-sample for a single species (*C*. *lathami*) is shown for both the Ion Torrent and MiSeq in Fig 3B and 3C a similar error profile was found across all species on both platforms.

When dealing with amplicon sequencing, determining not only the overall error rate for the target region but also calculating an error spectrum can have many benefits. In doing this, certain error “hot-spots” can be detected, and being aware of the presence of such areas can enable more informed decisions in relation to determining OTUs, calling SNPs and verifying taxonomic identifications. Having a good understanding of the composition of the chosen target region can also be of benefit. If the area of the amplicon that proves to be most informative is at the 3’ end of the amplicon sequence for instance, it is possible to optimally position the direction of sequencing. The profile may also dictate if a paired end strategy is more appropriate. Single-source samples specific to the targeted gene region can also facilitate the monitoring of run-to-run variation in error rates specifically for the amplicon of choice.

Awareness of the error profile and composition of an amplicon gene region is an important consideration that can impact upon one’s ability to taxonomically discriminate taxa. If an amplicon sequencing approach is adopted some of the biases associated with PCR and primer skews may also be minimised, or can at least be highlighted, by ensuring that the primer binds on all taxa of interest through the use of *in silico* bioinformatics [71]. It is also worth being aware of the fact that no primer is truly universal. It is therefore worthwhile to consider the use of a multi-locus approach especially given the current patchy state of reference databases where some taxa may be present for one gene region but not another [72,73]. Lastly, it is worth noting that just because a primer set is said to “work well” in one study (or because it is a currently accepted DNA barcode) it does not necessarily follow that it will also be fit for purpose in another study. This issue is clearly highlighted in the case of Australian mammals where the conventional barcode COI is wholly insufficient due to the poor representation of Australian marsupials and rodents for this gene in current databases such as GenBank or BOLD [24,72,74].

### 3.3. Experiment 3: Importance of experimental controls

Once an appropriate target region(s) is selected and DNA extracts are screened for copy number and inhibition, decisions then turn to how best to build a library free of artefacts and contamination. The issue of contamination and artefact formation should always be considered when PCR is involved. Amplicon sequencing on 454, Illumina or Ion Torrent, always involves the manipulation of PCR products, thus workflows are susceptible to contamination. Amplicon sequencing workflows on current second generation platforms involve multiple rounds of PCR [75,76], many published workflows utilise three rounds of PCR [77–80]: a primary PCR, an MID (Multiplex Identifier) tagging PCR (i.e. indexing) and then amplification within emulsions (454, Ion Torrent) or on a flow cell (Illumina). Unlike Sanger sequencing when low-level contaminants presented as a ‘bumpy’ baseline, HTS will show these as unambiguous sequences. In many respects high-throughput amplicon sequencing should be viewed as the “white-glove” test of laboratory cleanliness.

A major potential source of contamination is due to the handling of amplicon products post-PCR. Thus it is strongly recommended (where possible) to conduct pre-PCR and post-PCR work in independent, dedicated spaces or labs, preferably physically separated form each other. It is advisable to minimise the handling of untagged amplicon products as much as possible to prevent cross-contamination of samples. It is for this reason that methods such as nested- or hemi-nested PCR, reamplification, and ligation of ‘sequencing adapter-MID tag’ sequences to untagged amplicons can be problematic. Employing nested-PCR approaches to enrich for low abundant taxa may be more prone to contamination and/or artefactual sequences when compared to PCR-free targeted enrichment of amplicons.

It goes without saying that minimising contamination is essential in all studies where amplicon sequencing is used, especially those that seek to explore diversity in instances where it arises as a result of low-abundant taxa or variants [81,82]. The increased sequencing depth afforded by HTS should not be viewed as a means by which to “cut-through” potential contamination be it environmentally derived or otherwise. This is particularly true in scenarios where endogenous DNA is highly degraded or in low copy number, as is the case for ancient or environmental DNA, where modern or well-preserved DNA sequences will amplify more readily. The degree to which a sample has been contaminated cannot be known *a priori* and such contamination, especially environmentally derived, may not always be low-level. Increased sequencing depth, therefore, will do nothing to dilute the level of contaminant sequences, and neither will arbitrary cut-offs designed to remove low-abundant unique sequence clusters or OTUs. There is no substitute for environmental, extraction and PCR blank reaction controls. The failure to use controls can never be justified and nor can the failure to report the use of controls, even when they turn up negative results. Controls are the only true means by which it can be determined whether or not the fidelity of samples have been maintained throughout processing. Controls are seldom reported in papers using HTS [83], especially in the fields of environmental DNA and microbial metagenomics. The lack of reporting of controls in bacterial metagenomics studies is alarming given the ubiquitous nature of bacteria. In the absence of such controls it is impossible to say what bacteria are endogenous to the samples collected or even the extent to which bacteria common to the environment contribute to the microbiome from which the sample was collected. This is particularly true when dealing with coarse taxonomic assignments at an ordinal or family level, not to mention when making claims about the presence, absence and/or abundance of OTUs. The importance of controls in bacterial metagenomics is clearly shown when considering that after OTU sequences present in control reactions conducted during bacterial profiling of hairs [37] were removed the number of OTU sequences present in scalp hair samples dropped by ~60–70% (S2 Table). Moreover, it is clear that this is not a simple case of PCR contamination arising from poor lab practice as the drop off for pubic hair, conducted within the same PCR plate was much lower at ~30% (see S2 Table and [37] for further details and also [84] for another example of using controls to filter sequences for contamination). High-throughput sequencing serves to hold up a magnifying glass to the laboratory practices of any lab that makes use of it. The depth at which a sample can be sequenced can result in even the lowest levels of contamination being revealed. This can be problematic where analyses and conclusions rely on low abundant sequences and the only assured means of retaining confidence in results and conclusions in these cases is through careful library preparation and considered data analysis. While it is easy to pick out common laboratory contaminants or aberrant sequences when such amplicons assign taxonomically to taxa not found in the study area, it is more difficult to account for cross-sample, environmental or laboratory contamination that closely resembles the taxa or sequence variants of interest.

The use of indexed (or MID tagged) primer sequences is not only useful in allowing the processing of multiple samples in parallel but it is also a convenient means by which to filter. This can be achieved by only allowing amplicon sequences with the exact MID tag to be used in further analyses. However, the use of the word “unique,” and other related terms, with respect to these MID tags is slightly misleading as in reality MID tags are often recycled across many samples. This may prove problematic due to sample carry-over that is observed with some platforms or potential library contamination by means of aerosolised particles during library generation. The issues surrounding the possibility of sample carry-over is best illustrated when considering the first Ion Torrent PGM run that the authors of this paper outsourced to a sequencing facility where, when the data was analysed, 25 tags not used in the preparation of the amplicon library were detected, amounting to 0.02% of the total number of reads returned. Out of these 25 tags, if the tag that was present in the greatest abundance had been used in the experiment, approximately 1.2% of the reads belonging to the sample to which it was assigned could have been indistinguishable contamination. In this instance it was clear that the contamination might have arisen at the sequencing facility itself as none of the tags detected were ever used in the laboratory where the amplicon library was generated. This highlights an important issue when considering the outsourcing of DNA sequencing to other labs, commercial or otherwise. It may be necessary in future to provide statistics of run-to-run carry over and the timeframe between the re-use of tags when such a sequencing facility also generates the amplicon for sequencing. Numerous studies are now beginning to highlight the issue of contamination arising from the laboratory, reagents and commercial kits [82,85]. Anecdotally, researchers also talk about contaminating data from sequencing facilities but it is rarely, if ever, reported in the literature.

A simple strategy to limit issues associated with this is to increase the timeframe between the first use and subsequent re-use of an MID tag. While it is tempting when dealing with a small number of core loci to re-use a limited number of tags, such as those officially released by the platform manufacturers, it nonetheless increases the likelihood of contamination creeping in from run to run and building up over time. Expanding the number of MID tags used in a lab greatly reduces the potential of MID tag contamination with little extra cost. A further means of ensuring tag contamination is kept to a minimum is the use of differing MID tags at the 5’ and 3’ end of the amplicon sequences (see Section 2.1.2), which can also benefit in terms of data filtering to increase the likelihood of only high quality sequences being retained. Additionally, the use of different 5’ and 3’ MID tags on an amplicon greatly increases the number of possible combinations at a laboratory’s disposal. Finally, the use of different 5’ and 3’ MID tagged amplicons may also help in the detection of chimeric sequences. The downside of a method such as this however is the cost associated with ordering primers; although this can be kept to a minimum by not ordering HPLC purified primers as synthesis errors are easily managed by post-run filtering. Moreover quality control validation by mass spectrometry is now commonplace and serves to minimise the likelihood of primers with high proportions of incorrect bases.

While some might argue that the purchase of MID tagged primers is expensive the counter argument is that so too is repeating runs where the researcher believes the data is compromised. In our lab six reads were detected of a Chinese herbal plant from one study [36] that turned up in a palaeosediment sample from Australia. In this instance both samples shared the same MID tags despite being many runs apart. In sensitive applications the re-use of MID tags may be a false economy. Low-template samples necessitate sensitivity and single-use of tag combinations. This has the added benefit that each amplicon product generated is unique to the originating sample and contamination can be removed bioinformatically.

### 3.4. Experiment 4: Library generation efficiency

The opening and closing of PCR-tubes or plates post-PCR and the handling of untagged amplicon products serve to increase the chances of untraceable contamination as a result of poor laboratory technique or the release of aerosolised amplicons. It is for this reason that a single “full” fusion tagged TSP (see S1E Fig) PCR approach [21,86] or sequencing adapter ligation post-MID tagging [27] via PCR method is preferable from the perspective of contamination control. The drawbacks associated with a “full” fusion tagged TSP PCR approach centre around a loss of PCR efficiency due to the long fusion primers required and also the problems surrounding primer-dimer. However, careful size selection can assist with dimer removal [87–90]. The ligation of sequencing adapters post-MID tagging via PCR itself can be inefficient and may be biased towards the preferential ligation of certain amplicons or terminal bases. In some cases the efficiency drop-off associated with a “full” fusion tagged TSP approach can be mitigated through the use of the modular tagging of amplicons using a single PCR (MoTASP) method [21] or by simply spiking in some standard non-fusion TSP into the PCR reaction containing “full” fusion tagged TSP (see S1E). The latter showed generally modest efficiency improvements when compared to qPCR in the absence of spiking in standard non-fusion TSP, however the C_T_ value shifts in qPCR varied considerably for each platform (S3 Table). Additionally, the spiking in of standard non-fusion TSP when using “full” fusion tagged TSP still showed a general increase in C_T_ values when compared to qPCR containing only standard non-fusion TSP, particularly in the case of the MiSeq (S3 Table). Although the MoTASP method has been reported to improve PCR efficiency, it is unclear as to the extent this may be the case as qPCR was not carried out and neither was a direct comparison of sequencing results [21].

The use of a “full” fusion tagged TSP approach where a library is generated in a single step is theoretically the cleanest way to generate amplicon libraries. The downside to this is the drop in PCR efficiency discussed above. A common alternative pathway is a series of primary PCRs which are pooled and followed by a secondary PCR to amplify sequencing adapters and/or MID tags onto the target sequences. Notwithstanding the contamination risk inherent to this two-step approach it is also the source of inter-sample chimeras, presumably through incomplete extension and/or ‘jumping’ PCR [91]. Practitioners need to carefully weigh the benefits and drawbacks of each library building method and be cognisant of how the method impacts on the conclusions they hope to draw from the resultant data.

### 3.5. Experiment 5: Analysis parameters and their impact

It is beyond the scope of this study to delve into the complexities of data analysis. It is however relevant to note that amplicon data can be analysed in many different ways, sometimes subtly so, that can result in quite dissimilar outcomes. It is also worth noting that analysis parameters are contingent on the benchwork component of amplicon sequencing workflows. To date there is no currently accepted best practice pipeline or approach to the analysis of amplicon sequencing output, although many do exist [28,42,92,93]. Nevertheless one of the few agreements on the way in which both shotgun and amplicon sequencing data is handled is the necessity to filter sequences for error and potential contamination in a manner that strikes a balance between overly relaxed and unnecessarily stringent filtering. The manner in which such filtering is done and the definitions associated with various processes along the filtering pipeline can have a marked impact on the final result. Naturally, the stringency and type of filtering method employed is both platform dependent and sensitive to the library building methodology.

**Fig 4:**
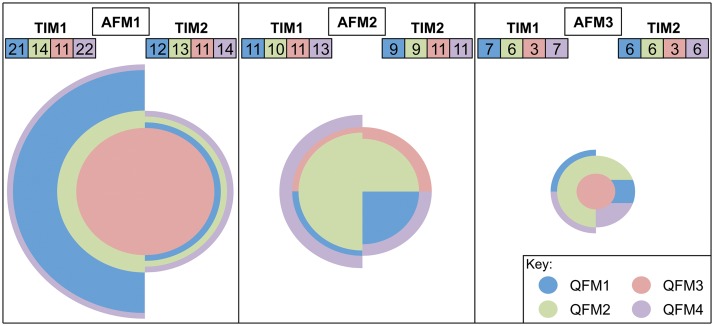
Impact of analysis parameters on the numbers of taxonomic units obtained for a bulk-bone sample. A number of analysis parameters were used to analyse a complex mixture containing numerous taxa. Different quality and abundance filtering methods were used in addition to two taxonomy-independent measures of analysis, full definitions and explanations of which are in Fig 1. The spread in the numbers of taxonomic units obtained across the combinations of parameters chosen is seen. The radius of each semicircle represents the number of taxonomic units obtained given a set combination of the parameters used. The number of taxonomic units is also indicated above each semicircle. Each semicircle is proportional to all others. AFM—abundance filtering method; QFM—quality filtering method; TIM—taxonomy-independent method.

The difficulty of analysing the diversity of samples whilst accounting for sequence quality, abundance and attempting a taxonomy-independent measure of analysis is illustrated in Fig 4. Depending on the quality filtering method (QFM), abundance filtering method (AFM) and taxonomy-independent method (TIM) used (Figs 1 and 4) the number of taxonomic units detected varied between 3 and 22 operational taxonomic units (OTUs) or between 3 and 14 distance-based operational taxonomic units (DTUs) [24] (Fig 4). In each case the minimum average Quality Scores (Q-Scores) for all sequences post-filtering were well above the standard cut-off of Q15. Tellingly however, when considering QFM1 and QFM4 (see Fig 1 for definitions and also S4 Table) where individual bases below Q15 were permissible, a sizeable proportion of sequences contained bases below Q15 (57.0% and 42.3% respectively) and there was a noticeable percentage of bases below Q15 overall (2.6% and 0.9% respectively) (S4 Table).

The use of Phred Q-Scores, as noted above, is one means by which to filter sequence data for error. Many papers, including those by the authors, make mention of how the data contained within has been filtered for quality, however, few make mention of how this is done thus making it difficult to reproduce data from the pipeline used. It is an open question as to what truly constitutes a high quality sequence. For instance, is it one where the average Q-Score across its length is >Q20 or should it be a requirement that all bases within the sequence be at least Q15? Q-scores are also complicated by the fact that different platforms use different methods when generating Q-scores. An issue surrounding the use of a stringent Q-Score cut-off that all bases must meet is the fact that the Q-Score of a base is impacted by the Q-Scores of the bases immediately surrounding it. Homopolymers are generally areas of quite low quality and this low quality can extend for a number of bases beyond the homopolymer stretch itself. In an extreme example, a Q-score based filtering method might actively discard amplicon variants that contain homopolymer stretches in favour of those that do not, thereby warping the composition of the resultant data.

In addition to Q-score cut-offs, filtering of sequence reads below a certain abundance is often employed. This is often cited as an attempt to reduce the possibility of erroneous and artefactual sequences as well as to remove instances of low-level contamination. At times such an approach could be seen as the molecular biology equivalent of “sweeping the dirt under the carpet”—simply moving a baseline until one is happy with the data will ultimately reduce sensitivity and reduce transparency of data fidelity. As with Q-score quality filtering, abundance filtering can be performed in a variety of ways with no accepted definition of what should be classed as a low abundant grouping of sequences. Methods of abundance filtering vary from the removal of singletons only, to the use of, at times, arbitrary cut-offs or inferred cut-offs defining a low abundance cluster (see Fig 1 for examples and Fig 4 for impacts). The choice of an appropriate abundance filter is no easy task especially in cases where there is unequal sequencing depth that may necessitate the need for sample specific abundance filters.

The fluidity of the definition of a high quality sequence and what constitutes a low abundance cluster as well as the order in which filtering steps are performed (see Fig 1 for examples and Fig 4 for impact) can all combine to create a rather difficult analysis of the diversity of a sample when dealing with heterogeneous samples of unknown composition. This holds true not only when dealing with the abundance of sample constituents but also when dealing with presence and/or absence. These factors are exacerbated further when weighted analyses are employed. In reality there is no means by which to determine the “correct” number of OTUs within a sample. For instance, with regards to a pool of single-source bird samples containing a single sample of only one representative of the family Dromaiidae, *Dromaius novaehollandiae* (Emu), a total of four distinct OTUs were obtained post-filtering (data available from authors upon request). Also worth noting is the importance of ensuring samples are free of inhibition and have sufficient copy number of DNA when conducting OTU analyses that involves a requirement for a particular OTU to occur in a certain proportion of uniquely tagged replicates before it is accepted [94]. If such a criterion were used in the two-fish screening assay (Fig 2), the genus *Engraulis* would have been excluded at times as it only occurred in a single replicate in certain cases, even though its presence was confirmed using *Engraulis* specific primers. Notwithstanding the above, when used appropriately, OTUs can be a useful index for species diversity provided parameters are both transparent and consistent across samples and studies.

## Conclusion

It is proving to be the case in amplicon sequencing that a one-size-fits-all approach is ill-advised and unwise, due to differing budgets, scopes and end-goals. It is therefore not the aim of this article to call for definitive guidelines with regard to best practice when generating amplicon libraries or sequencing them, although a set of flexible reporting guidelines may be appropriate. It is hoped that this paper may instead prove to be a catalyst ultimately aiding in the development of robust amplicon sequencing workflows. The generation of amplicon data is easy, however the generation of high-fidelity data free of contamination, artefacts and appropriately analysed, is far more complex. It is important to be aware of the limitations of amplicon data and know that with the advances afforded by it there are many hurdles. It is imperative that more attention be paid to the processes involved in preparing amplicon libraries to limit some of the pitfalls highlighted in this paper. While published data can be analysed and re-analysed time and again, such as when reference databases improve, the library generation step is not as easily, quickly or cheaply repeated. It is widely acknowledged that amplicon sequencing will continue to play an important role across a wide range of applications. Taken together these data suggest that, in order to get the most out of amplicon datasets, careful attention should be paid to workflows at both benchtop and desktop.

## Supporting Information

S1 Fig Schematics of the steps involved in each of the experiments performed. (PDF)

S1 File Single-source bird error output example. (XLS)

S2 File DTU calculation example. (XLS)

S1 Table Primer information. (PDF)

S2 Table Proportion of sequences removed post control filtering. (PDF)

S3 Table Cycle threshold value shifts when performing fusion tagged PCR. (PDF)

S4 Table Summary quality statistics. (PDF)
