# Supplementary material for: From Benchtop to Desktop: Important Considerations when Designing Amplicon Sequencing Workflows
Source: PLoS One. 2015 Apr 22;10(4):e0124671. doi: 10.1371/journal.pone.0124671 (PMC4406758; doi:10.1371/journal.pone.0124671)
Supplement: S1 Fig — (PDF) [file pone.0124671.s001.pdf]

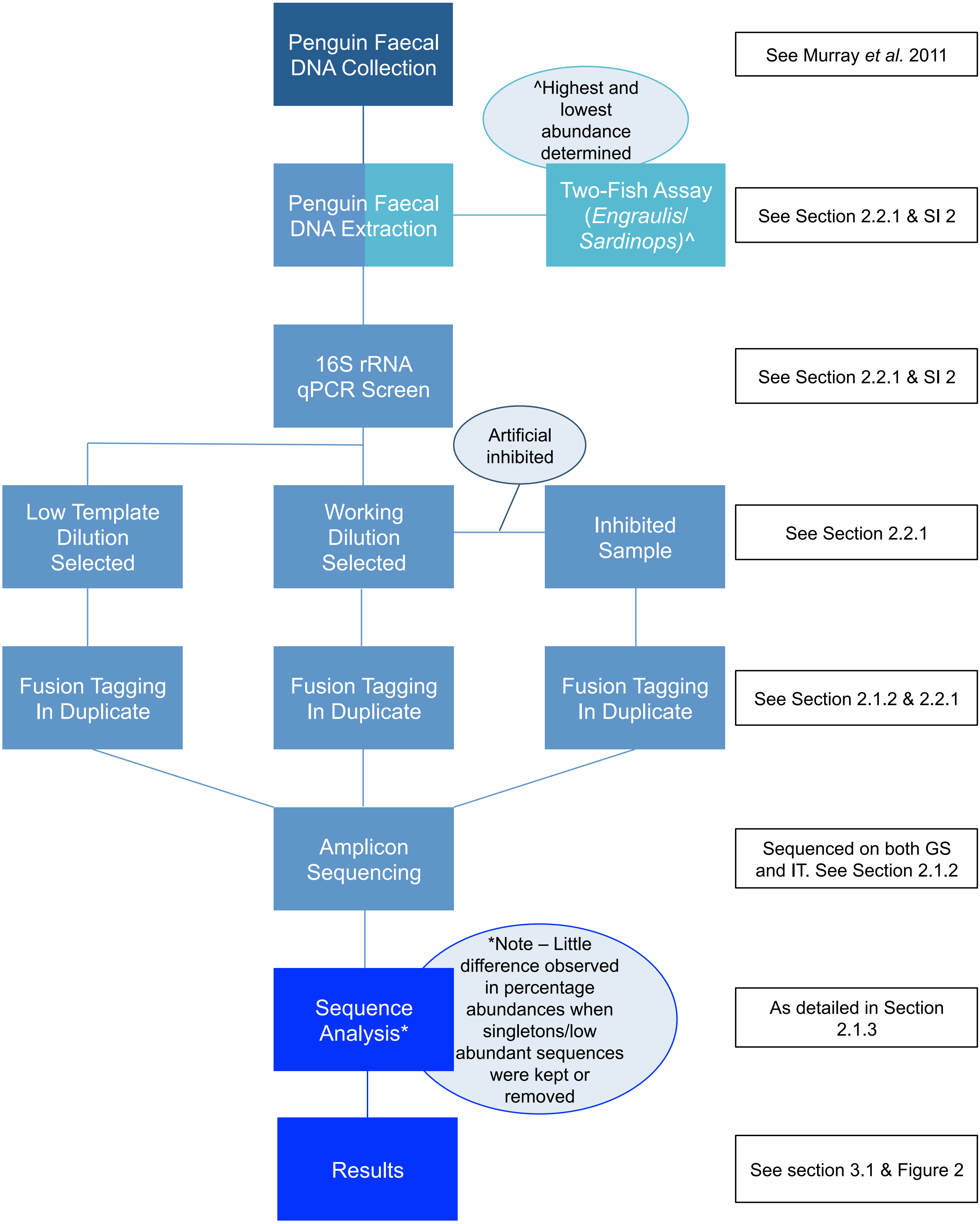

Figure S1A. Experiment 1: Importance of sample screening. Schematic showing steps involved in the experiment determining the impact of inhibition and low template amount on the successful detection of two fish genera

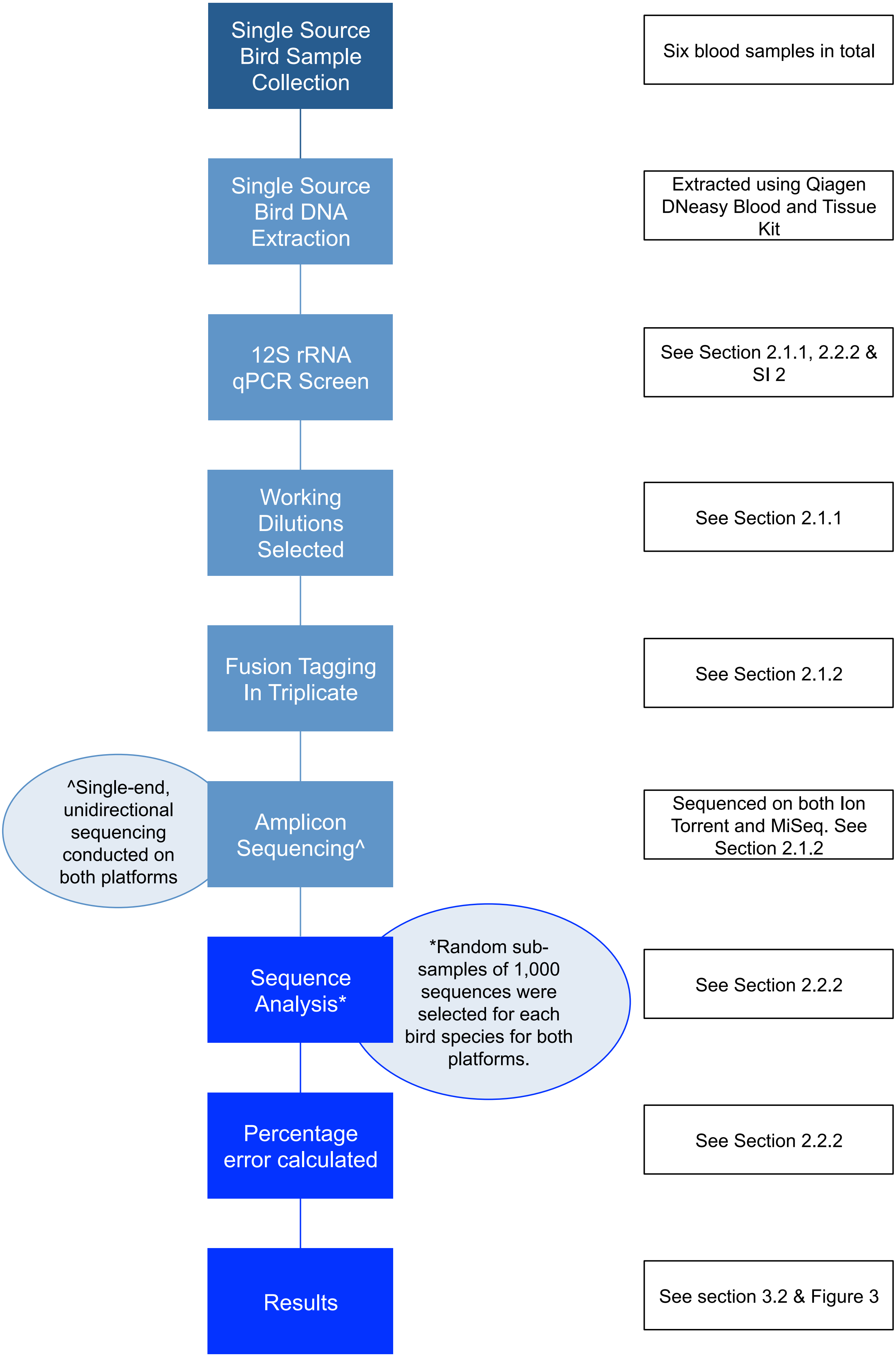

Figure S1B. Experiment 2: Assessing the amplicon target region. Schematic showing steps involved in the experiment illustrating the benefits of characterising and understanding the target region in amplicon sequencing

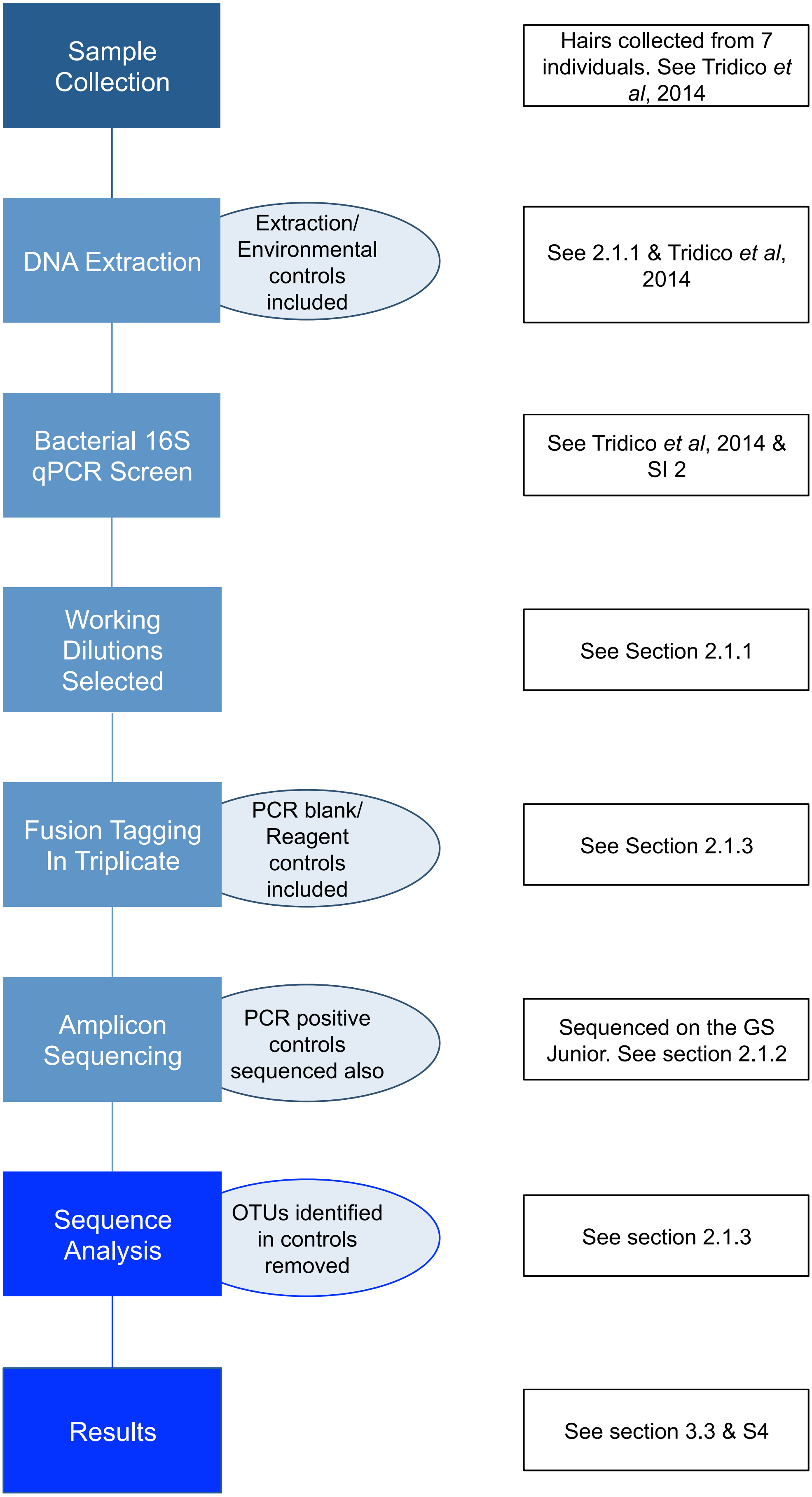

Figure S1C. Experiment 3: Importance of experimental controls. Schematic showing steps involved in the experiment illustrating the importance of controls along each step during the preparation of amplicon libraries

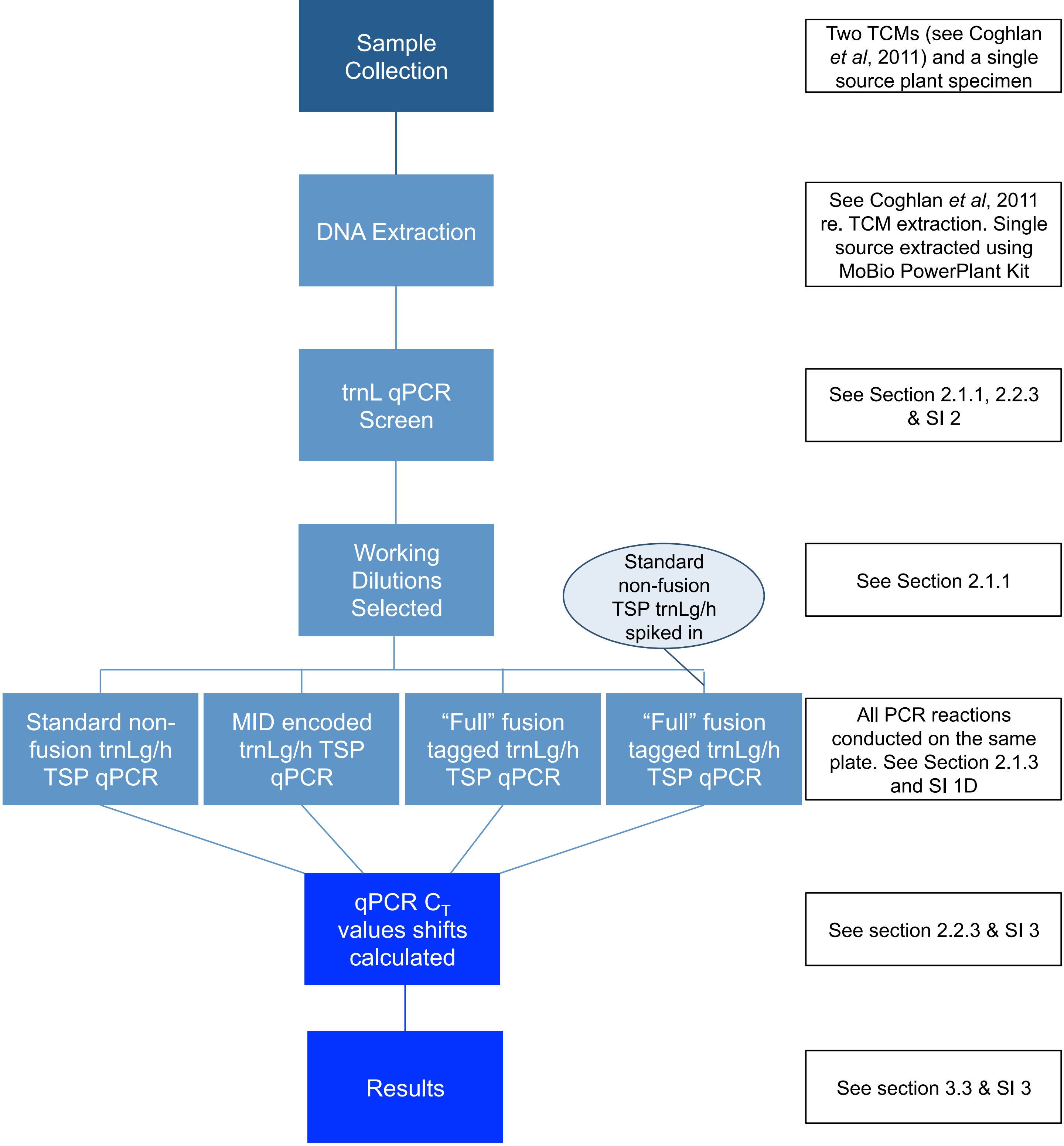

Figure S1D. Experiment 4: Library generation efficiency. Schematic showing steps involved in the experiment assessing the reduced efficiency of PCR amplicon generation due to long fusion-tagged primers and the amelioration of.

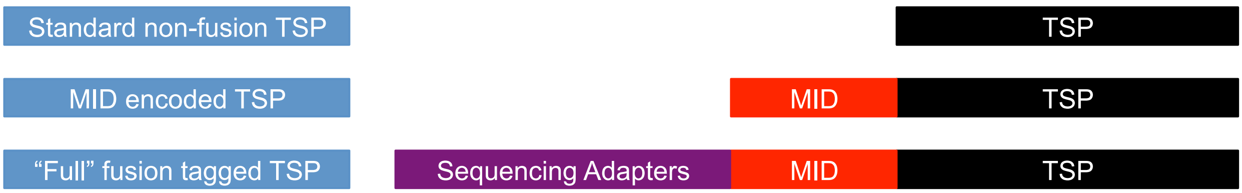

Figure S1E. Primer Architecture. Diagram showing the architecture of the primers used in experiments. TSP – Template specific sequence (e.g. trnLg primer); MID – Multiplex Identifier Tag (i.e. unique DNA index); Sequencing Adapters – Platform specific adapters required for clustering (MiSeq) and/or sequencing (all platforms).

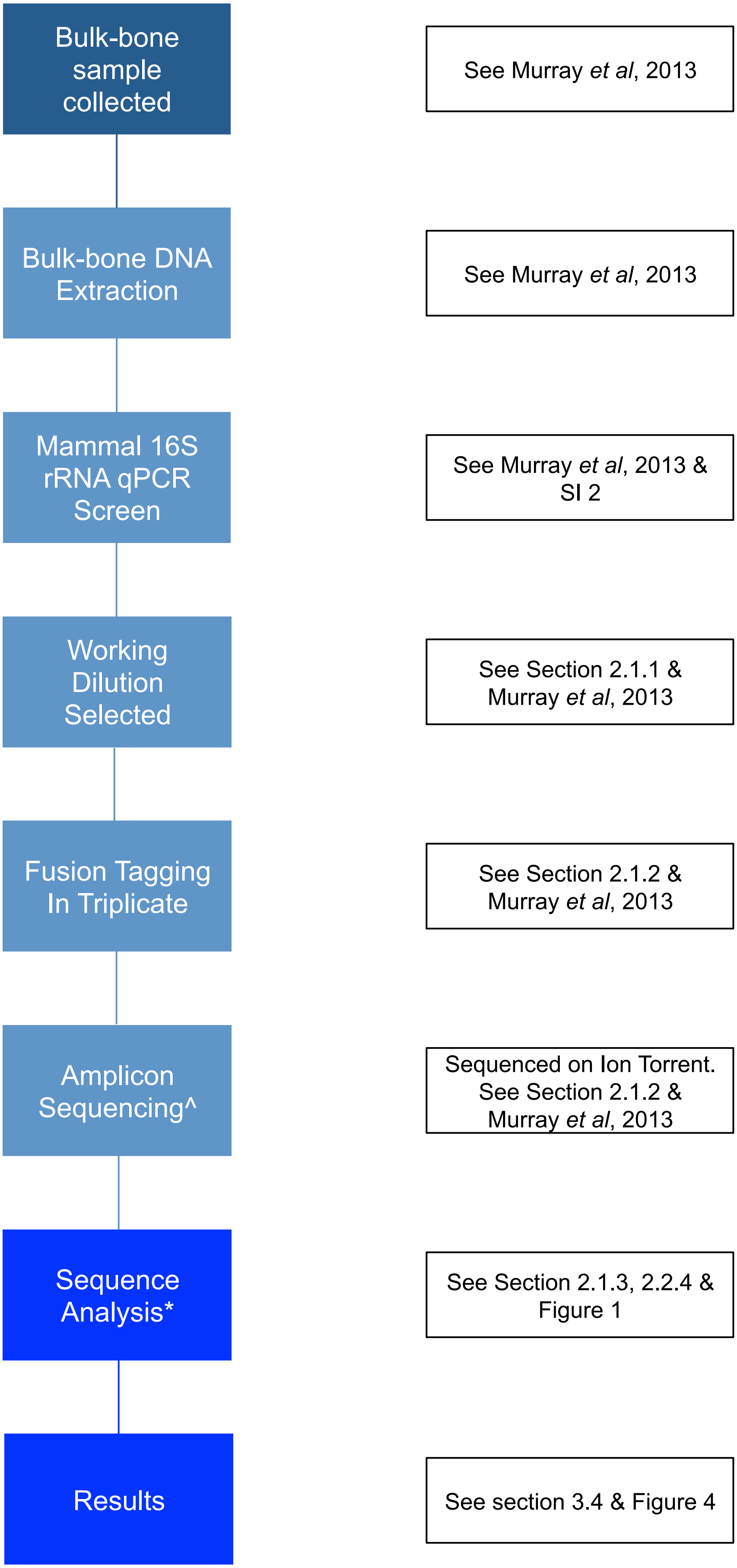

Figure S1F. Experiment 5: Analysis parameters and their impact. Schematic showing steps involved in the experiment illustrating how choosing different analysis parameters can impact greatly on the number of taxonomic units determined to be in a sample.
