# Supplementary material for: From Benchtop to Desktop: Important Considerations when Designing Amplicon Sequencing Workflows
Source: PLoS One. 2015 Apr 22;10(4):e0124671. doi: 10.1371/journal.pone.0124671 (PMC4406758; doi:10.1371/journal.pone.0124671)
Supplement: S1 Table — (PDF) [file pone.0124671.s004.pdf]

Table S1. Primer Information

Primer name	Target Taxa	Sequence (5'-3')	Annealing temp. (ºC)	Experiment	Reference
16S1F-degenerate<br>16S2R-degenerate	Fish	GACGAKAAGACCCTA<br>CGCTGTTATCCCTADRGTAACT	54	1	1
PIL1F<br>PIL1R	*Sardinops sagax*<br>(Australian Pilchard)	CCTAACTGGAGCCCCAAAC<br>GCTGTGGCTCTGGGTTTTAG	60	1	2
AN1F<br>AN2R	*Engraulis australis*<br>(Australian Anchovy)	CCTAAATACCCGCAGCCTTAT<br>CAACTCTCGGCTTAAGGGTTT	60	1	2
16Smam1<br>16Smam2	Mammals	CGGTTGGGGTGACCTCGGA<br>GCTGTTATCCCTAGGGTAACT	55	5	3
12SA<br>12SH	Aves	CTGGGATTAGATACCCCACTAT<br>CCTTGACCTGTCTTGTTAGC	57	2	4<br>5
Bact_16S_F515<br>Bact_16S_R806	Bacteria	GTGCCAGCMGCCGCGGTAA<br>GGACTACHVGGGTWTCTAAT	54	4	6<br>7
trnLg<br>trnLh	Plants	GGGCAATCCTGAGCCAA<br>CCATTGAGTCTCTGCACCTATC	52	3	8

Details are provided for each primer set used in this paper including the sequence, annealing temperature and taxa targeted. Additionally, the experiment in which each primer was used is given.

1. Deagle BE, Gales NJ, Evans K, Jarman SN, Robinson S, et al. (2007) Studying Seabird Diet through Genetic Analysis of Faeces: A Case Study on Macaroni Penguins (Eudyptes chrysolophus). PLoS ONE 2: e831.

2. Murray D, Bunce M, Cannell BL, Oliver R, Houston J, et al. (2011) DNA-based faecal dietary analysis: A comparison of qPCR and High Throughput Sequencing approaches. PLoS One 6: e25776.

3. Taylor PG (1996) Reproducibility of ancient DNA sequences from extinct Pleistocene fauna. Molecular Biology and Evolution 13: 283-285.

4. Cooper A (1994) DNA from Museum Specimens. In: Herrmann B, Hummel S, editors. Ancient DNA: Springer New York. pp. 149-165.

5. Cooper A, Lalueza-Fox C, Anderson S, Rambaut A, Austin J, et al. (2001) Complete mitochondrial genome sequences of two extinct moas clarify ratite evolution. Nature 409: 704-707.

6. Turner S, Pryer KM, Miao VP, Palmer JD (1999) Investigating deep phylogenetic relationships among cyanobacteria and plastids by small subunit rRNA sequence analysis. J Eukaryot Microbiol 46: 327-338.

7. Caporaso JG, Lauber CL, Walters WA, Berg-Lyons D, Lozupone CA, et al. (2011) Global patterns of 16S rRNA diversity at a depth of millions of sequences per sample. Proceedings of the National Academy of Sciences 108: 4516-4522.

8. Taberlet P, Coissac E, Pompanon F, Gielly L, Miquel C, et al. (2007) Power and limitations of the chloroplast trnL (UAA) intron for plant DNA barcoding. Nucleic Acids Research 35: e14.
