# Supplementary material for: From Benchtop to Desktop: Important Considerations when Designing Amplicon Sequencing Workflows
Source: PLoS One. 2015 Apr 22;10(4):e0124671. doi: 10.1371/journal.pone.0124671 (PMC4406758; doi:10.1371/journal.pone.0124671)
Supplement: S2 Table — (PDF) [file pone.0124671.s005.pdf]

Table S2. Proportion of sequences removed post control filtering.

Sample	Number of Sequences Without Control Filtering	Number of Sequences With Control Filtering	% of Sequences Remaining After Control Filtering
Female Scalp Hair	19762	6420	32.5
Female Pubic Hair	26714	18802	70.4
Male Scalp Hair	15776	6881	43.6
Male Pubic Hair	17514	12618	72.0

The following table shows the proportion of bacterial amplicon sequences lost after filtering operational taxonomic units from samples that were also found to be in DNA extraction and PCR negative controls.
