# Supplementary material for: From Benchtop to Desktop: Important Considerations when Designing Amplicon Sequencing Workflows
Source: PLoS One. 2015 Apr 22;10(4):e0124671. doi: 10.1371/journal.pone.0124671 (PMC4406758; doi:10.1371/journal.pone.0124671)
Supplement: S3 Table — (PDF) [file pone.0124671.s006.pdf]

Table S3. Cycle threshold value shifts when performing fusion-tagged PCR.

			(1)	(2)	GS (3)	GS (4)	IT (3)	IT (4)	MS (3)	MS (4)
Sample	Single Source Plant	Avg. C_T	25.63	25.75	28.21	27.61	26.65	26.40	30.61	27.96
		Diff. in C_T 1		0.12	2.58	1.98	1.02	0.78	4.98	2.33
		Diff. in C_T 2			-0.60		-0.24		-2.65	
	CAM Plant Screen 1	Avg. C_T	22.13	22.57	23.94	23.63	23.77	22.88	25.72	25.65
		Diff. in C_T 1		0.44	1.80	1.50	1.63	0.75	3.59	3.52
		Diff. in C_T 2			-0.31		-0.88		-0.07	
	CAM Plant Screen 2	Avg. C_T	21.37	20.76	22.46	22.50	22.79	21.77	26.00	23.76
		Diff. in C_T 1		-1.37	0.33	0.37	0.66	-0.37	3.87	1.63
		Diff. in C_T 2			0.04		-1.03		-2.24	

Diff. in C_T 1	Difference in C_T value compared to 1
Diff. in C_T 2	Difference in C_T value between 3 and 4

Cycle threshold values are shown for quantitative PCR reactions using one of the following: (1) - Standard non-fusion TSP; (2) - MID encoded TSP; (3) - “Full” fusion tagged TSP; (4) - “Full” fusion tagged TSP with standard non-fusion TSP spiked in (for further clarification see Section 2.2.4 of main article and S1 E Fig.) For (3) and (4) TSP sequences specific for each of the GS-Junior (GS), IonTorrent (IT) and MiSeq (MS) were used. Any the efficiency drop off associated with using "full" fusion tagged primers (3) when compared to standard non-fusion TSP (1) is shown as is whether any efficiency drop-off can be ameliorated using a spike in of standard non-fusion TSP when using "full" fusion tagged TSP (4).
