# Supplementary material for: From Benchtop to Desktop: Important Considerations when Designing Amplicon Sequencing Workflows
Source: PLoS One. 2015 Apr 22;10(4):e0124671. doi: 10.1371/journal.pone.0124671 (PMC4406758; doi:10.1371/journal.pone.0124671)
Supplement: S4 Table — (PDF) [file pone.0124671.s007.pdf]

Table S4. Summary Quality Statistics.

	QFM1	QFM4
Minimum Average Q-Score of a Sequence	18	27
Minimum Q-Score for a Base	4	5
% of Sequences with a Base of Q-Score ≤ 15	65.37%	54.1%
% of Bases with Q-Score ≤ 15	3.2%	1.3%

Table showing quality statistics of amplicon sequences resulting from quality filtering methods QFM1 and QFM4. Quality statistics were calculated post quality filtering prior to any further abundance filtering.

QFM1 – No mismatches in primer sequence allowed and no Q-score filtering; QFM4 – 2 mismatches in primer sequence allowed and USEARCH maximum expected error of 0.5.
